# Barriers to seeking post-abortion care in Paktika Province, Afghanistan: a qualitative study of clients and community members

**DOI:** 10.1186/s12905-021-01529-5

**Published:** 2021-11-06

**Authors:** Shiromi M. Perera, Haroon Achakzai, Monica M. Giuffrida, Meghana Jayne Kulkarni, Devin C. Nagle, Mohammad Kameen Wali, Sara E. Casey

**Affiliations:** 1 International Medical Corps, 1313 L St. NW, Suite 110, Washington, DC 20005 USA; 2 International Medical Corps Afghanistan, House # 11, Street-6, District -10, Qala-e-Fatullah, Kabul, Afghanistan; 3 RAISE Initiative, Heilbrunn Department of Population and Family Health, Mailman School of Public Health, Columbia University, 60 Haven Ave, New York, NY B210032 USA

**Keywords:** Post-abortion care, Afghanistan, Reproductive health, Barriers

## Abstract

**Background:**

Unsafe abortion is a leading cause of maternal mortality. In Afghanistan, which has experienced decades of armed conflict and where abortion is highly restricted, maternal mortality is high at 638 maternal deaths per 100,000 live births. Post-abortion care (PAC) is a lifesaving package of interventions to reduce morbidity and mortality related to induced or spontaneous abortion, but is rarely provided and often of poor quality, particularly in humanitarian settings. In July 2018, we conducted a study to identify the factors that influence access to and use of PAC services at Sharana Provincial Hospital.

**Methods:**

In-depth interviews (IDIs) were conducted with ten women who had received PAC services at Sharana Hospital, and eight focus group discussions (FGDs) were conducted with 40 married women and 40 married men aged 18–45 from four villages surrounding Sharana Hospital.

**Results:**

PAC clients and community participants discussed similar barriers to seeking PAC, including cost, distance to the health facility, the need for male accompaniment to seek care, perceived and actual quality of care, stigma and shame. Despite the mentioned stigma around abortion, community members expressed willingness to help women to receive PAC.

**Conclusions:**

Our results suggest that while some barriers are not unique to PAC, others, especially those related to stigma around abortion, may be specific to PAC. It is important for the Ministry of Public Health and its partners to prioritize addressing these barriers to ensure that women have access to this critical life-saving care.

**Supplementary Information:**

The online version contains supplementary material available at 10.1186/s12905-021-01529-5.

## Background

Afghanistan has one of the highest maternal mortality ratios in the world, with an estimated 638 maternal deaths per 100,000 live births [1]. Afghanistan has undergone a series of armed conflicts over the past four decades, widely decimating the health infrastructure during extended periods of war [2]. In the past two decades, following the overthrow of the Taliban regime, some improvements to maternal mortality indicators have been made, but there is still a significant disparity between urban and rural areas, with rural areas having markedly higher maternal mortality [3].

Complications of spontaneous or induced abortion are the fourth leading direct cause of maternal mortality globally, accounting for 7.9% of maternal deaths, an underestimation due to a lack of reporting and misclassification of abortion-related deaths [4]. In Afghanistan, safe abortion is legally permitted only to save the life of the woman or if the child will be born with a disability [5]. Although data on abortion incidence in Afghanistan are lacking, the abortion rate in the South-Central Asia region was 46 abortions per 1000 women aged 15–49 years old from 2015 to 2019; over half of abortions in the region were unsafe [6, 7]. In countries where abortion is permitted only to save the life of the woman or prohibited altogether, 75% of abortions are unsafe [7]. Unsafe abortions are also likely to increase in countries affected by war as a result of the breakdown of social and structural support systems that often accompany conflict [8].

Women in countries with restrictive abortion laws may be less likely to seek care for complications of abortion [9]. Post-abortion care (PAC) is a package of interventions that has been shown to reduce abortion-related morbidity and mortality when available and of good quality. PAC includes the following components which may be provided by mid-level providers in health centers: treatment of complications of induced and spontaneous abortion (miscarriage), counseling to identify and respond to a woman’s health needs, provision of contraceptive and other sexual and reproductive health services, and community mobilization to increase awareness and address PAC-related misconceptions and stigma [10–12].

Despite the lifesaving nature of PAC, these services are rarely provided, and often of poor quality in many countries; this situation is exacerbated in humanitarian settings [13–16]. In a 2010 cross-sectional study of emergency obstetric and newborn care (EmONC) in health facilities in Afghanistan, investigators found mixed availability of supplies for PAC: 70% of facilities had manual vacuum aspirators (MVA) and flexible cannulas; 88% of facilities had uterotonics, 97% carried essential antibiotics, and 94% had modern contraceptives [17]. However, the training of healthcare professionals was inadequate and knowledge regarding the most common complications of unsafe abortion, steps needed for the treatment of complications and provision of post-abortion contraception, was incomplete [17]. Among providers completing a knowledge assessment as part of the EmONC study, average knowledge scores for treating complications of incomplete abortion and what information to give PAC clients were 60% or lower–lower than they received on any other EmONC service [18]. In 2017, the Afghanistan Ministry of Public Health (MoPH) introduced Post Abortion Care Clinical Service Guidelines [5]; these guidelines have not yet been fully implemented across the country.

International Medical Corps (IMC) has provided life-saving health care, including maternal and newborn health services to tens of thousands of people affected by Afghanistan’s ongoing conflict in Nuristan and Paktika provinces. This includes capacity building of health workers and development of long-term capacity of supported facilities, as well as strengthening community education related to health and availability of services. Paktika, located in eastern Afghanistan, is a primarily rural province which has been heavily affected by the conflict [19]. For example, the province has the second highest number of damaged or closed schools [20]. The total fertility rate is 5.3, with 25.9% of women reporting a current pregnancy. The median age of first birth is 21.6 years, and 35.8% of births in the previous 5 years took place in a health facility [21]. Among currently married women aged 15–49, 28.9% reported current use of a contraceptive method, the majority of whom used lactational amenorrhea method; 20.3% reported an unmet need for contraception [21].

In partnership with the MoPH, IMC implemented the Basic Package of Health Services (BPHS) and Essential Package of Hospital Services (EPHS) in Paktika from 2004 to 2015, and only EPHS (supporting Sharana Hospital, the referral hospital for Paktika province) from 2015 to 2018 to provide life-saving emergency healthcare, including PAC services [22]. Sharana is a public hospital providing free services to patients. In July 2018, IMC, in collaboration with the Reproductive Health Access, Information and Services in Emergencies (RAISE) Initiative at Columbia University, conducted a mixed methods study to identify the factors that influence access to, use and provision of PAC services at Sharana Hospital. This manuscript focuses on two components of this larger study and results related to barriers to PAC use.

## Methods

In-depth interviews (IDIs) were conducted with ten women who had received PAC at Sharana Hospital in the 3 months preceding data collection. PAC clients who had a uterine evacuation received a brief description of the study from a hospital staff member while recovering after treatment and were asked if they would be willing to speak to an interviewer about their experience and the care they received. From the list of those PAC clients who agreed to participate, participants were purposively selected to represent a range of ages and contacted by program or facility staff, who then coordinated a date and time for the interview to be conducted at Sharana Hospital.

Eight focus group discussions (FGDs) were conducted with 80 married women and men aged 18–45 from four villages around Sharana, recruited by IMC staff: two with women aged 18–24 (n = 20), two with women aged 25–45 (n = 20), two with men aged 18–29 (n = 20) and two with men aged 30–45 (n = 20).

Semi-structured interview guides were developed separately for the IDIs and FGDs, adapted from tools used by RAISE in other countries (Additional file 1). The IDI guides covered topics including the decision to seek care and perceptions of the PAC received. Following a few broad questions about how the client was treated during her visit, the interviewer then probed on specific components of quality such as confidentiality, cleanliness, comfort, privacy, pain management and provider attitudes if these were not previously mentioned. FGD guides included the following themes: perceptions of women with spontaneous abortions and care-seeking behavior for spontaneous abortion, reasons why a woman would induce abortion, community perceptions of women who induce abortion and care-seeking behavior after an induced abortion. IMC recruited female and male interviewers with prior experience conducting qualitative research and who were comfortable discussing reproductive health topics. They completed a 5-day training on qualitative data collection methods, research ethics, discussion of abortion and values clarification activities to consider biases related to abortion. They conducted the interviews and discussions in Pashto, with female interviewers conducting all female FGDs and PAC client interviews and male interviewers conducting male FGDs. Data collection took place July 2–5, 2018. IDIs and FGDs were audio-recorded, transcribed in Pashto and translated into English for analysis by Afghan research team members fluent in both languages. Identifying information was removed during transcription. Researchers performed an initial review of transcripts to check quality and requested clarification when needed. IDIs ranged from 30 to 45 min and FGDs from 60 to 100 min.

### Analysis

The English transcripts of the IDIs and FGDs were reviewed with Pashto-speaking members of the research team. Researchers read the transcripts and created separate codebooks for the IDIs and FGDs with the major themes. After codebooks were finalized, transcripts and codebooks were uploaded to NVivo (v12), and all transcripts were coded independently by two researchers. When discrepancies arose, they were discussed and resolved until inter-coder agreement was established. After coding was completed, researchers used thematic content analysis to identify the main themes related to barriers. Barriers discussed in FGDs and IDIs were compared to look for commonalities and differences in community perceptions and PAC clients’ personal experiences. The standards for reporting qualitative research (SRQR) guideline was used for reporting this study (Additional file 2).

### Ethical considerations

To protect confidentiality, all discussions were held in a private space and no names were included in transcripts. Verbal informed consent was obtained from all participants; no remuneration was provided for participation. Only members of the research team had access to the recordings and transcripts. Ethical approvals for the study were obtained from the Institutional Review Boards of Columbia University and the Afghanistan National Public Health Institute.

## Results

### Participant demographics

A total of 10 IDIs were conducted with PAC clients at Sharana Hospital. Participants’ ages ranged from 18 to 35 years and the number of lifetime pregnancies ranged from 2 to 14 (Table 1). All respondents were married and reported that they had no formal education. They all reported that their abortions were spontaneous, not induced.

**Table 1:** Socio-demographic background and reproductive history of PAC client in-depth interview respondents (n = 10), Sharana Hospital, Paktika province, 2018

	N
*Age*	
18–24	3
25–35	5
Unknown	2
*Marital status*	
Married	10
*Education level*	
No formal education	10
*Gestational age*	
Less than 13 weeks	8
13 weeks or more	2
*No. of pregnancies*	
1–3	3
4–6	4
7 or more	3

Focus group participants (n = 80) were male and female community members between 18 and 45 years old (Table 2).

**Table 2:** Focus group participants (n = 80), Paktika province, 2018

Age and sex	N
18–24 years, married women	20
25–45 years, married women	20
18–29 years, married men	20
30–45 years, married men	20

PAC clients discussed their own decision-making to seek care and their experiences with PAC at Sharana Hospital, and FGD participants discussed community perceptions of abortion and post-abortion care-seeking. Although the PAC clients are women who succeeded in seeking PAC, they and focus group participants discussed numerous barriers that may prevent or delay women from seeking PAC. Several broad themes emerged during analysis, and the results were organized according to these thematic areas, including delays in care-seeking, perceptions of quality of care, and shame and stigma surrounding abortion.

### Delays in care-seeking

Some IDI respondents reported no delay in care-seeking, going to a health facility shortly after feeling pain, bleeding, or weakness from abortion-related symptoms. However, other IDI respondents experienced delays in care-seeking due to a variety of reasons, including lack of transport, lack of recognition that their symptoms required treatment and concerns about cost. Some mistakenly believed that the hospital would be closed due to the public holidays such as Eid and Ramadan. For some, these barriers led to life-threatening complications including losing consciousness due to severe blood loss.

#### Distance

Sharana Hospital was far from home for most of the PAC clients interviewed and required travel by motorcycle or a vehicle, a barrier for some.

Yes, I started bleeding. Two days went by until I came to the hospital. …We do not have any car or anything. We live far away [~20km]... Therefore, by the time we were getting here, the time had passed. (PAC client, 25–35 years)

It’s very far away [~20km]. We don’t have a motorcycle nor do we have a car. Someone else brought us here. We are living far away in the mountains. (PAC client, 25–35 years)

The long distance may have played a role in clients seeking PAC at other facilities before coming to Sharana Hospital. The majority of women sought care at a clinic or pharmacy before presenting at Sharana. Community members in the FGDs also mentioned the distance to facilities that provided PAC as a barrier for women who experienced abortion.

Oftentimes this issue is happening in remote areas of the province like in some districts in which people don’t have access to health facility centers. But people like us who reside in the center area of the province have no problems because we have access to health facilities like hospitals and clinics. And when our females have problems, we take them to the doctor. (Man aged 18–29)

They do not [seek care]. There is no male, no car, and if someone lends them the car then they are delivered [to the health facility]. If not then they are left like that and they die. (Woman aged 25–45)

#### Male accompaniment

All IDI respondents reported traveling to the hospital with their husband or other male relative (usually a brother-in-law). Some women mentioned that they felt uncomfortable discussing their symptoms with other male family members if their husband was not present.

My brother-in-law came with me. My husband has not been with me because he has been doing hard manual labor work. (PAC client, 18–24 years)

My bleeding got severe and I had very bad pain in my belly. My husband told me right away that let’s go to the hospital. (PAC client, 25–35 years)

Several participants from the FGDs described that women may face challenges seeking PAC if they did not have husbands or male relatives to accompany them to the facility, with one participant saying that the doctor would not treat a woman if her husband were not present.

We are being abused in the hospital as well. The doctors of the hospital are telling us not to come to the hospital alone [without a male]. There has to be a man with you when coming to the hospital. The doctors are telling us ‘you are a newly married wife, why are you coming to the hospital without any man?’ (Woman aged 18–24)

Some female FGD participants expressed that women should be able to attend facilities alone, despite the existing cultural barriers.

…this is the culture of the society, but it shouldn’t be. If a woman [is] faced with difficulties and her males [are not] with her, she should go to the hospital and nobody should look at her in a [negative] way. (Woman aged 18–25)

#### Cost of PAC services: experience versus perception

Most of the interviewed PAC clients reported receiving the services for free or at low cost at Sharana Hospital. (Sharana is a public hospital and services are supposed to be provided at no cost to the patient.)

I think two or three thousand (US$25–40) were spent. Other things didn’t cost. Neither did the tests cost nor the [ultrasound], nothing… it wasn’t a lot. It was a fair amount. (PAC client, unknown age)

Payments for costs such as medications, transport to the facility, and food represented a significant burden for some PAC clients and their families. One woman first described the cost of services as too much, then later said the cost was worth the end result of her health and recovery.

We bought the medicines, and we bought things to eat which helps control low blood pressure. …I think three or four thousand Afghani (US$40–50) was spent out of my husband’s pocket…We are poor people so even that amount is a lot of money for us. We borrowed it from someone. (PAC client, 25–35 years)

The health workers provided some medications, but clients were also expected to purchase some medications (including pain medications) from a local pharmacy or bazaar.

Here, they give the medicine either by the hospital or…they write us the prescription to buy it for 1000 or 2000 (US$13–26) from outside. (PAC client, 25–35 years)

…We bought all the medications from the bazaar, we have not been given medicines from the hospital. When we visit them the doctor prescribes and orders us to take this medication from a private pharmacy. (PAC client, 18–24 years)

FGD participants commonly perceived cost as a barrier to seeking PAC services. Participants said that women and their husbands could be deterred from visiting a health facility due to concerns about the cost of care and treatment.

After miscarriage, I told my husband to take me to the hospital because I am having complications. [He] told me ‘you are going to recover at home, no need for going to the doctor’....Because he didn’t have any money. (Woman aged 18–25)

…Most of the family will have economical problem when they take her to the doctor, they spend a high amount of money on her in order to get healthy. (Man aged 18–29)

### Clients’ perceptions of quality of care at the health facility

#### Hearsay reported about Sharana Hospital prior to seeking care

PAC clients discussed what they had heard about Sharana Hospital before coming for care, often negative things about how the health workers treated women in the hospital.

From all those who had come here to Sharana from our village. They would say that ‘they [health workers] don’t behave well with patients. There is no respect, no privacy, and the women just lie down without having any privacy.’ (PAC client, unknown age)

If someone is giving them a bribe then they have good attention towards those, but the poor ones just lie there. (PAC client, 25–35 years)

Other women reported that they had heard good things about the health facility prior to attending.

We used to hear it from anyone else’s mouth. They would suggest us to go to the hospital because there have been capable and experienced doctors in the hospital, and there in the hospital women are given quality medicines. (PAC client, 18–24 years)

#### Lack of suitable providers of PAC

FGD respondents questioned whether those providing PAC in local health facilities had adequate capacities and training to do so. Male FGD participants, in particular, discussed the lack of female health workers as a barrier to PAC seeking, suggesting they would instead seek medicines with unknown efficacy from private pharmacies.

However, they didn’t take their women to the hospital due to a lack of female doctors. Therefore, their husband takes medicine from the private pharmacy by his own desire and then they give that to their wife... (Man aged 18–29)

### Perceptions of quality of PAC services received

Despite largely negative information heard about the hospital prior to coming, PAC clients were generally satisfied with the quality of the PAC they received at the facility. The majority of clients believed the health workers treated them with respect and provided good medical care; however some reported negative experiences. Nearly all women reported they thought Sharana health workers would keep their information private.

I believe that they will keep it [my information] private and they are not going to share it with anyone else. (PAC client, 18–24 years)

I am so happy with them. I had such a day full of respect. They were walking around checking on patients, and I am happy from them. I would say that I don’t think there could be better doctors anywhere else than the ones here. (PAC client, unknown age)

Others reported negative experiences with privacy.

No, no, there is no privacy at all. … First they lay you down on the bed and then all staff are standing around. Recently when I was getting a service and when they put on me the machine all of them were standing in a line from here to over there and were watching. I really felt ashamed that time. (PAC client, 18–24 years)

Now they don’t hang any curtains or anything. The patients are just lying down there. (PAC client, unknown age)

Some clients indicated they did not receive adequate information from the health workers. In some cases, their husbands or other family members received information, perhaps due to the severity of their condition and lack of consciousness during treatment. However, health workers may not have made every effort to explain sufficiently the complications, treatment and follow-up necessary to the patient herself. One woman specifically mentioned that the female doctor was more clear with her than the male doctor.

They didn’t speak to me; they spoke with my husband. (PAC client, unknown age)

I didn’t know what the male doctor was saying, but the way female doctors talked with me was really clear. They instructed me on the dosages of the medications, then they gave me their phone numbers. (PAC client, 25–35 years)

Clients reported mixed experiences with pain management: some were satisfied while others were unhappy with how their pain was managed during treatment and recovery.

… At night I told them my back has been paining badly, they told me “it doesn’t matter because we have just cleaned you, you are going to recover slowly”. (PAC client, 18–24 years)

While some clients reported good cleanliness, others were less satisfied.

There was a girl who had just delivered a baby, and after her they made me lie down on [the same bed]. (PAC client, 25–35 years)

### Shame and stigma related to abortion

Many FGD participants expressed negative attitudes towards both induced and spontaneous abortion. According to some participants in all FGDs, induced abortion was considered a sin in the Islamic faith. No matter the size of their family, participants believed God would provide for them.

… Basically, if a woman has been pregnant and ends her pregnancy it is big sin, ... If a woman has diseases and problems, producing a baby may cause her to die, sharia gives them permission [to end the pregnancy]. Then it doesn’t matter; but not for the reason that I have lots of children and I may not be able to provide them food because food is given by Allah. The more people in the world increase, the more Allah increases their food. (Man aged 30–45)

Strong negative views about women who induce abortion emerged early in the discussions, including that women who induce abortion are committing murder, and are generally bad people.

All people know that losing a child is assassination in this world or killing a baby is killing humanity. (Man aged 30–45)

That she has aborted it by her own will and when she does not take permission from her family then people look at her as a bad person. They ask her “why did you do this?” (Woman aged 25–45)

Although participants expressed stronger negative views towards women with induced abortions, they also discussed blame and shame towards women who had spontaneous abortions.

Currently, most people should deliver children and nobody wants to lose any, but if the woman loses her baby, then she will be faced with family problems hundred percent because, her father-in-law and her mother-in-law will tell her ‘why didn’t you take care of yourself?’ (Men aged 18–29)

When they have a miscarriage then they are poorly treated. Some are not treated well by their husbands and neither by their in-laws. (Women aged 25–45)

When discussing barriers to care-seeking for either induced or spontaneous abortions, some participants mentioned that women may be ashamed to seek PAC due to the negative connotation of abortion in society or out of desire to keep the abortion a secret.

Most of the women abort their children secretly, and people aren’t willing to [let] their women [go to the] health center alone or without her husband’s permission.…If a woman goes to the health center alone or without her husband, then the family members may ask her ‘where did you go?’ (Men aged 18–29)

Most of the women are afraid of their families, so they don’t reveal their problem and [keep it a] secret and hide things. Those who don’t get scared of their family then they eventually reveal their secret. (Woman aged 25–45)

Although some FGD participants suggested that family members may not help a woman seek PAC if she had induced the abortion, participants in all FGDs largely supported PAC for women experiencing complications even of induced abortions so that they do not die. Male participants also discussed their support for PAC in order to avoid additional costs related to treatment of more severe complications. A few groups mentioned the high costs of marrying a new wife if their current wife was perceived as incapable of producing children as a result of the abortion or, as one mentioned, if she was to die of complications.

We should help her, but not to share ourselves in her sin. (Woman aged 18–24)

If someone find[s] out about it then they do not [allow her to die], they help her at that moment. Later, after her recovery they don’t behave well with her...they ask this woman why did you do this? (Woman aged 25–45)

She goes to a clinic…we don’t let a woman die because she has already terminated [the pregnancy] so why should a woman be ruined, so we take her to the doctor, clinic, and we buy medication for her. (Man aged 30–45)

## Discussion

More than three decades of conflict in Afghanistan has resulted in severe damage to the country’s health infrastructure and services. The findings from this study suggest that while the barriers to access and use of services are not specific to the conflict and some are not unique to PAC, others, especially those related to stigma around abortion, may be specific to PAC. Study participants discussed a variety of barriers to seeking PAC, including cost and distance, male accompaniment, perceived and actual quality of care provided by the health workers or hospital, stigma, and shame. These barriers are likely to be even higher in other areas where PAC services received no targeted support.

Cost, even when the services were purportedly free of charge, as well as distance from the facility, were cited as both real and potential barriers to seeking PAC. Several clients reported paying for services at Sharana Hospital, where PAC services should be provided for free according to MoPH policy. Although corruption in the Afghan health system is extensive [23], most references to cost of services in our study appeared related to the need to purchase medications outside the health facility rather than informal requests for payment. It is important that Sharana Hospital address the supply chain problems causing these stockouts and ensure that health workers do not request formal or informal payments for PAC services. Evidence from other studies shows that the cost of health services can act as a barrier, particularly if women do not earn income or control their own finances as is often the case in Paktika [24, 25]. Even when the services are free, indirect costs such as transportation, medicine, or food can act as deterrents to seeking care, and disproportionately affect the lowest income households [23, 26]. Distance increases the cost of transportation to a health facility, is associated with reduced health facility utilization, and can be particularly challenging in a rural and insecure setting like Paktika province [27–29]. It is possible that drivers may charge more for transport due to the increased risk when traveling to or from areas affected by insecurity. Out-of-pocket spending on health in Afghanistan is high, representing 78% of total health expenditure in 2018 [23, 30]. Consequently payment for services, including medications, has been shown to be a main barrier to service utilization for rural families who generally have lower incomes and cannot afford informal service fees and medicines [26]. Our findings related to cost as a barrier are consistent with other studies on maternal and newborn care-seeking in rural areas of Afghanistan [26, 31]. Reducing out of pocket expenditures and transportation costs have been shown to improve health facility utilization in other settings [32, 33]. It is important to not only address costs, but also to inform the community that services are free given the frequent mention of cost as a barrier in the FGDs even though the majority of PAC clients reported paying minimal or no fees. Increasing the number of health facilities that can provide basic EmONC, including PAC, by extending services to where mid-level providers are based, is one way to reduce distance barriers [12, 34]. Transport vouchers to mitigate travel costs have also been shown to improve facility attendance [34]. Community-based services that provide free emergency transport from homes to health facilities may also be effective in overcoming distance barriers and help women arrive at a facility more quickly [35].

All PAC clients reported visiting the facility with a male relative, and consulting with their husbands or in-laws before seeking care. This was supported by the FGDs, in which participants said that a doctor may refuse to provide treatment to an unaccompanied woman. In a study of births in Pakistan, obtaining permission from one’s husband to go to a health facility was a common barrier to institutional delivery, particularly in rural areas [36]. In Afghanistan, women’s autonomy and ability to make decisions about health care are limited and constitute important barriers [37, 38]. While male partner involvement in abortion care has been shown to be positively associated with women’s well-being [39], in this context, the requirement for a man to accompany a woman is a barrier if a male relative is unwilling or unable to take her to the health facility [31, 40]. In addition to educating women about the need to seek PAC, it is necessary to work with communities to ensure men as decision-makers are targeted with this information so that women reach health facilities in emergencies, even when the male decision-maker is absent. In light of the rather transactional view of women expressed by some male participants, an economic argument can be used to encourage their support of early treatment. For example, messages could target men’s economic concerns by encouraging them to seek treatment early before complications become more expensive to address. Some women expressed reluctance to discuss their situation with a male relative, suggesting the importance of also educating female decision-makers, such as mothers-in-law. Finally, working with health workers to clarify the importance of treating women requiring emergency care, whether she is accompanied by a male relative or not, may also help to reduce these barriers to accessing PAC.

In many of the IDIs and a few FGDs, respondents reported hearing negative impressions (hearsay) of the care provided at the hospital, which evidence suggests causes women to avoid or delay seeking care [13, 41]. Perceived good quality of care, particularly observable structural elements of quality, such as number of providers and facility infrastructure, has been associated with higher utilization of obstetric care services [42, 43]. If a woman or her husband or family members believe that the health facility is dirty, or that the health worker is incompetent or disrespects them, they may hesitate or decide against going to the health facility, resulting in delays in care and poor health outcomes. It is important to recognize that news of one person’s negative experience can spread in the community. Supportive supervision is needed to ensure that quality of care is maintained. Stigma can also impact quality of care; in areas where abortion is stigmatized, women who perceived that health workers at the facility would mistreat them or violate their confidentiality reported reluctance to seek care [25]. PAC clients that have induced abortions may have a more negative personal experience compared to those coming in for spontaneous abortions. Values clarification and discussion of respectful care should be conducted with health workers at the hospital to ensure that non-judgmental good quality care is provided to all women.

In practice, many of the PAC clients in the IDIs expressed positive personal experiences with the health facility, indicating that the preconceived notions of the facility may have been incorrect. It could be helpful to engage with the community to dispel rumors of poor quality care, so that women or their families do not delay care-seeking for PAC. Specifically, community scorecards, which are used by community members, leaders, and health providers to monitor and assess publicly provided services, including health and sanitation services, have had some success in enhancing accountability and engaging health systems actors with community members globally and in Afghanistan, and may be a useful tool in this context to promote trust and increased utilization of health services [44].

All interviewed PAC clients reported that their abortions were spontaneous, so they may not have had the same experience of shame or discrimination from family and community members as have women who are known to have induced abortion. Many FGD participants discussed disapproval and stigma towards induced abortion and women who have them. Stigma around abortion persists globally, and can lead women to hide their abortion, seek an unsafe abortion and subsequently avoid seeking health services for complications [25, 45–47]. In other settings, community stigma around induced abortion is a barrier for access to quality healthcare services, particularly in rural settings [46]. It is, however, important to highlight that despite the strong negative reactions to induced abortion, participants in all FGDs said that a woman who induced abortion should still receive lifesaving PAC. Community disapproval towards induced abortion alongside community support for PAC has been documented in other settings [48, 49]. These results suggest that decreasing stigma related to both spontaneous and induced abortion are needed to ensure better and quicker access to PAC.

Community mobilization efforts to decrease stigma related to both spontaneous and induced abortion could increase access to PAC. Addressing abortion stigma as a barrier to PAC has been successful through interventions such as values clarification and attitude transformation (VCAT) workshops with health workers, community leaders and other stakeholders [50, 51]. Community health workers and community leaders could play a key role in engaging with their community to reduce abortion stigma and increase access to PAC.

### Limitations

It is important to consider the limitations to our study. We interviewed women who succeeded in receiving PAC and all clients reported a spontaneous abortion; women who failed to seek PAC or who induced abortion may experience additional barriers. It is possible that some women induced their abortion but reported it as spontaneous given the legal restrictions in Afghanistan. It is also possible that women who agreed to participate in interviews are those who had a more positive experience at the facility, or women may have demonstrated a courtesy bias to please interviewers. Another limitation is that some PAC clients appeared to contradict themselves over the course of their interviews when discussing quality of care. The research team engaged in detailed review of the full interviews to determine which opinion seemed to more accurately reflect their views for use in analysis. FGDs were conducted in communities located in government-controlled areas to ensure the safety of the data collectors and participants. Communities located in areas controlled by armed opposition groups may experience additional barriers. Facilitators did not probe on how insecurity impacted access. This research was conducted before the 2021 Taliban takeover of Afghanistan, and the changing context may potentially impact the findings and recommendations made here.

## Conclusion

Our results suggest that while some barriers are not unique to PAC, others, especially those related to abortion stigma, may be specific to PAC. Reducing indirect costs and ensuring services are free could increase utilization. It is important to improve the quality of care at the health facility so that perceptions of poor quality of services do not deter women from seeking care. Values clarification activities should also address stigma and bias among health workers to ensure respectful care. Community engagement, including with men, should address perceptions of the health facility, as well as reduce the spread of incorrect information about quality and cost of care. In addition, community engagement should address abortion stigma, among women, their families and their communities. However, it is important to remember that despite the barriers and negative perceptions of abortion in this community, community members expressed willingness to help women to receive PAC. It is important for the Ministry of Public Health and its partners to prioritize addressing the barriers to ensure that women have access to this critical life-saving care.

## Supplementary Information

**Additional file 1**. In-depth interview guide for PAC clients and Focus group guide.

**Additional file 2**. SRQR checklist

## Data Availability

The interview and focus group guides are included here as Additional file 1. The data used during the study are available from the corresponding author on reasonable request.

## Abbreviations

BPHS: Basic Package of Health Services
EPHS: Essential Package of Hospital Services
EmONC: Emergency obstetric and newborn care
FGD: Focus group discussion
IDI: In-depth interview
IMC: International Medical Corps
MoPH: Ministry of Public Health
MVA: Manual vacuum aspiration
NGO: Non-governmental organization
PAC: Post-abortion care
RAISE: Reproductive Health Access, Information and Services in Emergencies Initiative
VCAT: Values clarification and attitude transformation
