# Supplementary material for: Barriers to seeking post-abortion care in Paktika Province, Afghanistan: a qualitative study of clients and community members
Source: BMC Womens Health. 2021 Nov 6;21:390. doi: 10.1186/s12905-021-01529-5 (PMC8571834; doi:10.1186/s12905-021-01529-5)
Supplement: Supplementary file 1 — Additional file 1. In-depth interview guide for PAC clients and Focus group guide. [file 12905_2021_1529_MOESM1_ESM.pdf]

Additional file 1 - Study guides

In-depth interview guide for PAC clients

Selection criteria: PAC clients who had uterine evacuation (MVA, D&C or misoprostol)

Introduction

I want to thank you for taking the time to meet with me today.

Read the consent form

Do you have any questions about what I just explained?

Do you agree to participate in this interview?

How old are you?
What is the last year of school you completed?
Are you currently in school?
What is your marital status?
How many times have you been pregnant?

Decision to seek care

1. Thinking about *this most recent pregnancy*, at the time when you became pregnant, would you say that you wanted to become pregnant at that time?
   Probe: Were you trying to get pregnant? Where you trying to avoid it? How?

2. Thinking about this most recent pregnancy, how far along were you when it ended? When it ended, what happened?
   Probe: What was happening in your life at the time? Did the pregnancy end because of an action you took to end the pregnancy, or because of an accident in your body that had nothing to do with something you intentionally did?

3. What did you think when this happened? How did you feel?

4. Why did you think you needed to seek care? How did you know something was wrong?
   Probe: bleeding, pain, etc…

5. Did you talk about this problem with anyone else before deciding what to do?
   Probe: With whom? Husband? Family? Friends? What did they say? How were they involved in helping you through the problem?

6. Before coming to this facility, did you seek help for your problem from anyone else? From whom? What did this person do / what treatment did the person offer?
   Probe: did you go anywhere else or see anyone else before coming to the facility?

7. [IF SHE WENT ELSEWHERE FIRST] Why did you go to this/these place(s)? Why didn't you go to the health facility first?

8. Who suggested you come to this facility for your problem?

9. Why did you come to this facility for post-abortion care?
   a) Had you heard anything about PAC services provided at the facility? What did you hear?
      Probe: Friendly provider? Confidentiality? Cost? Free treatment?
   b) How did you hear this information or from whom?
      Probe : Community health workers, friends, family, providers
   c) Is this health facility the one nearest to your home? If not, why didn't you go to the facility nearest to your home?

10. Was it a difficult decision to come to the facility to seek care for your problem? Why or why not? Did you have any concerns about coming?
    Probe: cost, privacy, discrimination, pain, stigma, family planning, sterility

11. How did you get to the facility? Did anyone come with you? Who?

12. Do you hope to have another pregnancy? When would like your next pregnancy?

Post-abortion care received

13. How did you feel during your visit? Did you feel any pain during the procedure?
    Probe: cleanliness, privacy, comfort, pain management

14. How do you feel you were treated by the provider? Why? What did he/she do to make you feel this way?
    Probe: How the provider made her feel, could she understand the provider, did the provider take time to make client comfortable? Any judgmental or stigmatizing treatment?

15. Do you think the information that you shared with the provider and your reason for coming to the facility will be kept private? Why or why not?

16. Thinking about the information you received, was the information clear enough? Do you feel like all of your questions were answered? If not, what questions did/do you still have?
    (Explain that questions can be answered/ referred after the interview)

17. Did the provider explain that you could become pregnant again right away? Did he/she discuss family planning with you? Did you choose to use a method? Which method? Why did you choose this method?

18. [If she didn't choose a method]: Why didn't you choose to use a FP method?

19. Did you pay for any of the services you received during this visit? How much did you pay? What do you think of the amount you had to pay (too much, appropriate)?

Post-abortion Reflections

20. Overall, was your experience at the facility good or bad?

Probe: privacy, respect, pain, amount of time spent

21. Would you recommend that a friend or family member who bleeds during pregnancy come to this facility for post-abortion care? Why or why not? What would you tell a woman in your situation to do?

22. Was there something that could have been done differently that would have made you feel better about your experience? If yes, what?

23. [IF SHE SOUGHT CARE ELSEWHERE FIRST]: Would you have done something differently when you first realized you needed help with your problem?

24. Do you know anyone who was badly hurt or who died after an unsafe abortion? If yes, what advice would you give them now?

Thank you so much for your time! The information you have shared with us will be kept anonymous (your name will not be used) but will help us give feedback to our providers and improve our services for women.

Focus group guide for community members

Introduction
I want to thank you for taking the time to meet with me today.

READ CONSENT FORM.

Are there any questions about what I have just explained?

Are you willing to participate in this discussion?

Section 1: Spontaneous abortion
We are going to talk about women who have complications during pregnancy. What type of pregnancy complications do women experience here?
Probe: Abortions, are there are a lot?

If a woman bleeds during the first half of pregnancy, what is the reason for it?

In your community, how do people react to women who experience bleeding during the first months of a pregnancy (have a miscarriage, spontaneous abortion)? What do people think of her?
Probe: stigma, exclusion, discrimination, support, organized effort to respond to emergency needs, family planning

What does a woman do if she experiences bleeding during the first half of her pregnancy (miscarriage, spontaneous abortion)?

(Probe if going to a health facility is not mentioned): Have you heard of women going to a health facility with a problem after a spontaneous abortion? What do you think of that?

Why would a woman not go to a health facility if she has bleeding during the first half of her pregnancy (miscarriage, spontaneous abortion)? Women go to health facilities for other health problems, so why don't they go for bleeding during pregnancy? Why is it different?

Section 2: Induced abortion
Sometimes a woman becomes pregnant when she does not want to. What happens if a woman has a pregnancy when she is not ready?
Probe: Are there women who end a pregnancy for which they are not ready?

What ways do you know for women to end a pregnancy? Where do they get the method to do so? Who helps women end their pregnancy? Of the ways you mentioned, which are known to be the most effective?

How do women find out about methods to end a pregnancy? Is it something they discuss with their family? Friends? Health care provider?

Why would a woman try to end her pregnancy?

What do people in the community think about a woman who ended her pregnancy? Would it be different if the woman was young? Poor? If she were raped?
Probe: What do women think about it? What do men think about it?
Would people in your community accept a woman ending her pregnancy and support her? Would people in your community isolate her in any way or make her feel bad? What would they do? Why would they treat her in that way?
Probe: Try to find out about specific actions that community members might do to support or isolate, discriminate against the woman, her treat her badly. Do you have friends who have told you that they ended a pregnancy? Would men want to marry a woman who has ended a pregnancy?

Who in your community is the most affected by ending pregnancies?
Probe: Are there certain groups of women who are treated differently if they are suspected of having an abortion? For example, young women, poor women, women who have been raped? If yes, how are they treated differently?

What happens if something goes wrong after a woman has ended her pregnancy? What does that woman do?

(*Probe if going to a health facility is not mentioned):* Have you heard of women going to a health facility with a problem after an abortion? What do you think about that?

Why would a woman not go to a health facility if she has bleeding during the first half of her pregnancy? Women go to health facilities for other health problems, so why don't they go for bleeding during pregnancy? Why is it different?

Would you recommend that a woman go to a health facility if she has a problem after an abortion? Why or why not?

Wrap-up
Is there anything else that you would like to add or discuss now that you think is relevant to this issue?

Do you have any questions or concerns?

Thank you very much for your time.

*Use probes when needed:
- *Would you give me an example?*
- *Can you elaborate on that idea?*
- *Would you explain that further?*
- *I'm not sure I understand what you are saying.*
- *Is there anything else you'd like to share about that?*
- *Do you /others agree with that? What do you/ others think?*
- *I see some signs of doubt/ disagreement/ confusion. Can you explain?*

د جنین د سقط وروسته پاملرنی(PAC) د مراجعینو سره تفصیلي مرکه:

پیژندګلوي:

د هرڅه مخکي له تاسو نه مننه چي نن مو ماته مو وخت راکړي او له ما سره مو وکتل.

د رضایت دغه پاڼه ولولی

ایا د هغه څه په اړه چي ما اوس تشریح کړه کومې پوښتني شتون لري؟

ایا تاسو غواړي چي په دي بحث کي ګډون وکړي؟

څوکلنه یاست؟

څومره زده کړي مو کړي دي؟

اوس په ښونځي زده کړي کوي؟

څوځله امیندواره سوي یي؟

د مرستي غوښتلو پریکړه:

1 - ددي وروستي امیند واري په اړه فکر وکړی، کله چه میندواره شوی ایا تا غوښتل چه شی که نه ؟
تحقیق : ایا تا غوښتل چه میندواره شی که نه؟ ایا د منع لپاره دی هم کوشش کړی وو؟ که هو نو څنګه ؟

2 - ددی وروستی میندواری په اړه که فکر وکړی؛ د څومره وخت لپاره ستا په خیټه کی پاتی شوه؟ کله چه ختمه شوه، د هغی نه وروسته سه وشول؟
تحقیق: په هغه وخت کي ستاسو په ژوند کي څه حالت وو؟ آیا د امیندواری پای ته رسیدل د کومي کړني له امله رامینځه ته سول چي تاسو غوښتل امیندواري پاي ته ورسوي، یا ستاسو په بدن کي د کومي حادثې له امله پاي ته ورسیدل چي تاسو په قصدي توګه د هغه سرته رسول نه غوښتل؟

3 - تاسو څه فکر کوي کله چي دا پیښه وسوه؟ څه احساس مو کوي؟

4 - تاسو ولي دا فکر کوي چي تاسو صحی پاملرني ته اړتیا لرلي؟ تاسو څنګه پوه سوي چي کومه ستونزه سته؟
تحقیق: د ویني تلل، درد، او نور
...

5 - ایا تاسو د دې ستونزي په اړه د بل چا سره خبرې وکړي مخکي له دې چي پریکړه مو وکړه چي باید یو څه وکړي؟
تحقیق: د چا سره؟ خاوند؟ کورنی؟ ملګري؟ دوی څه ویل؟ دوی د دې ستونزي په وخت کي ستاسوسره څه ډول مرسته وکړه؟

6 - د ي مرکز ته د راتلو نه مخکي، ایا تاسو د کوم بل مرجع نه د مرستي غوښتنه وکړه؟ له چا څخه؟ دا کس څه کار کوي / هغه کس ددي ستونزي لپاره څه علاج در وښوي؟
تحقیق: ایا دي مرکزته د راتلو نه مخکي کوم بل ځاي ته تللي یي یا دي بل چاته مراجعه کړیده؟

7 - [که لمړي دا بل ځاي تللي وي] ولي لمړي هغه ځاي/ځایو ته ولاړي؟ ولي په لمړي سر کي روغتیایي مرکز ته نه تلي؟

8 - چا تاسو ته وړاندیز وکړ چي تاسو د دې ستونزي لپاره دي مرکز ته راشي؟

9 - د سقط نه وروسته د پاملرني د خدمتولپاره ولي دي مرکز ته راغلي؟

a) ايا تاسو په دي مرکز کي د PAC خدماتو چمتو کولو په اړه څه اوريدلي وه؟ څه مو اورېدلي وه؟
تحقيق: دوستانه خدمات؟ محرميت؟ لګښت؟ وړيا درملنه؟

b) تاسو دا معلومات څنګه و اوريدل؟ د چانه مو واوريدل؟
تحقيق: د ټولنې روغتيايي کارکونکي، ملګري، کورنی، روغتيايي کارکونکي

c) ايا دا روغتيا مرکز ستاسوکورته نږدې دې؟ که نه، ولې تاسو خپل د کورسره نژدې مرکز ته مراجعه نه کول؟

10 - ايا دا يوه ستونزمنه پريکړه وه چي ستاسو دخپل ستونزې د درملني لپاره دي مرکز ته راشي؟ ولې؟ ولي نه؟ آيا تاسو دلته د راتلو په اړه کومه انديښنه لرله؟
تحقيق: لګښت؟ محرميت؟ تبعيض؟ درد؟ فاميلی پلان؟ ويره؟

11 - دي مرکز ته څنکه راغلاست (په څشي کي راغلاست)؟ ايا ستاسو سره کوم څوک راغلي دي؟ څوک؟

12 - غواړي چي بيا امیندواره شي؟ کله غواړي بيا اميندواره شي؟

د جنين د سقط وروسته معالجه (پاملرنه):

13 - ستاسو د تدواى پرمهال مو څه احساس کوي؟ ايا تاسو د پروسيجر په جريان کي درد احساس کړی؟
تحقيق: پاکوالی، رازداري، آرامی، د درد اداره کول

14 - تاسو څه احساس کوي چي ستا سو سره ددي مرکز د معالجه څانګي د کارکونکی لخوا څه ډول چلند وسو؟ ولي؟ هغه/هغي څه وکړه چي تاسويي په اړه دا ډول احساس کوي؟
تحقيق: څرنګه صحی کارکونکی هغه ته دا احساس ورکړی؟ ايا هغه د صحی کارکونکی په خبره پوهيدله؟ ايا صحی کارکونکی ددی د هوساينی لپاره وخت واخيسته؟ ايا تا د صحی کارکونکی لخوا کم توپيری (ناسم) چلند وليده ؟

15 - فکر کوي هغه معلومات چي تاسو له صحی کارکونکی سره شريک کړی او ستاسو د راتګ لپاره ستاسو دلايل به محرم وساتل شي؟ ولي؟ ولي نه؟

16 - د هغه معلوماتو په اړه چي ترلاسه موکړه څه فکر کوي، ايا هغه معلومات په کافي ډول روښانه وه؟ ايا تاسو احساس کوي چي ستاسي ټولي پوښتنې ځواب شوي دي؟ که نه، تاسو تراوسه څه پوښتنې لري؟
(مخکي له مخکي تشريح کوي چې پوښتنې به د مرکې وروسته ځواب شي)

17 - ايا صحی کارکونکی درته وويل چي تاسو بيا هم اميندواره کيدای شي؟ ايا هغه د کورني تنظيم په اړه ستاسو سره خبرې وکړي؟ ايا تاسو د دوي کومه طريقه دځان لپاره غوره کړه؟ کومه طريقه ؟ ولي مو دا طريقه غوره کړه؟

18 - [که هغي کوم ميتود نوي غوره کړي] تاسو ولي دکورني پلان جوړني کوم ميتود د ځان لپاره غوره نه کړي؟

19 - ايا تاسو د خپلي درملني لپاره پيسي ورکړې؟ څومره پيسي مو ورکړې؟ څه فکر کوي ددي پيسو په اړه (ډيری وی او که مناسب قيمت يی وو)؟

د جنين د سقط وروسته غبرګونونه:

20 - په عمومي توګه، په دي مرکز کي ستاسو تجربه ښه وه يا بده؟
تحقيق: محرميت، درناوی، درد، څومره وخت يي ونيوي

21 - ایا تاسو به خپلو ملګرو او د کورني غړو ته دي مرکز ته د راتلو سپارښتنه وکړي تر څو د جنین د سقط وروسته د درملني لپاره دي مرکز ته مراجعه وکړي؟ ولې؟ ولې نه؟ که کومه ښځه ستاسو غوندي مشکل ولري تاسو به هغي ته څه وویاست؟

22 - ایا داسې کوم څه و چې کولی شو په بل ډول ترسره شوي وي ترڅوتاسوخپل تجربه په ښه توګه احساس کړی وي؟ که هو، څه؟

23 - [که یي لمړي بل ځاي کي درملنه کړي وي] ایا تاسو به کوم څه بل ډول ترسره کړي واي کله چی تاسو لومړی پوه شوي چې تاسو د خپلې ستونزې لپاره اړیني مرستې ته اړتیا لري؟

24 - ایا داسي څوک پیژني چي د غیرمسلکي جنین د سقط په پایله کي سخت ژوبل سوي وي یا یي ژوند د لاسه ورکړي وي؟ ستاسو پیشنهاد به څه وي هغی ته ؟

ستاسو څخه مننه چي موږ ته مو وخت راکړي! هغه مالومات چي موږ سره مو شریک کړه، محرم به وساتل شي (ستاسو نوم به ونه کارول شي) مګر دا معلومات او ځوابونه به زموږ سره مرسته وکړي ترڅو موږ خپلو کازکوونکو ت لازم هدایات ورکو او په خپلو روغتیایي مرکزو کي د ښځو لپاره په خپلو خدمتونو کي ښه والي راولو.

د گروپي بحث لارښود

(د رضایت دغه پانه ووایاست)

ایا د هغه څه په اړه چي ما اوس تشریح کړه کومې پوښتنې لرې؟

ایا تاسو غواړي چي په دې بحث کي ګډون وکړي؟

لومړۍ برخه: دجنین طبعی (غیر عمدی) سقط:

موږ د هغو ښځو په اړه خبرې کوو چي د امیندوارۍ په جریان کي اختلاطات او ستونزي لري. ښځینه دلته د امیندوارۍ په جریان کي کوم ډول اختلاطات او ستونزي تجربه کوي؟

تحقیق: د جنین سقط، ایا دلته ډیر دي؟

که چیرې یوه ښځه د امیندوارۍ په لومړۍ نیمایي کي ماشوم سقط (زیان) کړی ، نوعلت به یي څه وی ستا سو په نظر ؟

ستاسو په ټولنه کي، خلک د هغو ښځو په وړاندی څه عکس العمل لري چي د امیدوارۍ په لومړیو میاشتو کي د ماشوم سقط (زیان) تجربه کوي؟ خلک د هغې په اړه څه فکر کوي؟

تحقیق: د شرم احساس، جلا والی یا شړنه، تبعیض، ملاتړ، د بیړني اړتیاو د ځواب ویلو لپاره منظم کوښښ، کورني تنظیم (د ماشومانو تر منځ فاصله).

که یوه ښځه د امیندوارۍ په لومړیو میاشتو کي د ماشوم سقط (زیان) تجربه کوي، خپله هغه ښځه څه شی کوي ؟

تحقیق: [که چیرې روغتیایی مرکز ته تلل ذکر نشي]: آیا تاسو داسي ښځي پیژني چي د سقط وروسته روغتیایي مرکز ته ځي؟ تاسو د هغوی په اړه څه فکر کوئ؟

ولې شاید هغه ښځي چي د خپلې امیندوارۍ په لومړۍ میاشت کي ماشوم سقط (زیان) کوي روغتیایي مرکز ته لاړي نشي؟ ښځي د نورو روغتیایی ستونزو لپاره روغتیایي مر کزو ته ځي، نو ولې د امیندوارۍ په وخت کي د وینې دلاسه ورکولو لپاره نه ځي؟ دا ولې یو د بل سره توپیر لری؟

دوهمه برخه: دجنین اجباري سقط:

ځیني وختونه یوه ښځه نه غواړي امیندواره سي خو هغه امیندواره کیږي. په دي وخت کي څه کیږي که ښځه امیندواره سي خو هغه د امیندواره کیدو لپاره اماده نه وي؟

تحقیق: ایا داسي ښځي شته چي هغه حمل پای ته ورسوي د کوم لپاره چي دوی چمتو نوي؟

د ښځود امیندواریو د ختمولو لپاره کومې لارې پیژني؟ دوی چیرته دا کار ترسره کوي؟ څوک د میرمنو سره مرسته کوي ترڅو حمل پای ته ورسوي؟ د هغه لارو څخه چي تاسو یې یادونه وکړه، کومې یې تر ټولو اغېزمني بلل کیږي؟

میرمني د امیندوارۍ د پای ته رسولو لپاره میتودونه څرنګه پیدا کوي؟ ایا دا داسي څه دي چي دوی د هغه په اړه دخپلې کورنۍ، ملګرو، اویا د روغتیا یی کا رکوونکو سره پري بحث کولای سي؟

ولې به یو میرمن هڅه کوي یا غواړي چې امیندوارۍ پای ته ورسوي؟

د ټولني خلک د هغه ښځي په اړه څه فکر کوي کوم چي خپل حمل پای ته رسوي؟ آیا دا عکس العمل فرق کوي که ښځه ځوانه وي، غریبه وي او یا هم جنسي تیری پري شوی وي؟

تحقیق: ښځي د هغې په اړه څه فکر کوي؟ نارینه د هغې په اړه څه فکر کوي؟

آیا ستاسو په ټولنه کې هغه ښځه منی چې خپل امیندواری پای ته رسوی؟ آیا د دوی سره مرسته کوي؟ آیا ستاسو په ټولنه کې به خلک هغه د ټولني څخه په کوم ډول جلا کړي یا به هغی ته خراب احساس ورکړي؟ دوی به څه وکړي؟ ولې به دوی ورسره دا ډول چلند کوي؟

تحقیق: [هڅه وکړي چې د ځانګړو کړنو په اړه معلومات پیداکړي کوم چې د ټولني غړي یې ددي ډول ښځو سره کوي]: ایا ملاتړيي کوي یا یي بیلوي، د میرمنو په وړاندي تبعیض کوي، د هغو سره په ناوړه توګه چلند کوي. ایا تاسوکوم ملګري لري چې خپل حمل یي پای ته رسولی وي؟

ستاسو په ټولنه کې د حمل د پای ته رسیدو وروسته کوم ډول ښځی تر ټولو ستر زیان سره مخ کیږي؟

تحقیق: آیا داسي میرمني، داسي خاص ګروپونه یا ډلي شتون لري چې په دي وجه ورسره توپیري (متفاوت) چلند کیږي چي په هغو شک دي چي جنین یي سقط کړي؟ د بیلګي په توګه، ځواني ښځي، بې وزلې ښځي، هغه ښځي چې جنسي تیری پري شوي دي؟ که هو، دوی سره څنګه متفاوت چلند کیږي؟

که کومه ښځه د امیندواري د مینځه وړلو په وخت کې د کومي ستونزي سره مخ کیږي، نو بیا څه پیښیږي؟ دا ښځه څه کوي؟

(**تحقیق**: که چیرې روغتیایي مرکز ته تلل ذکر نشي): ایا داسي کومه ښځه پیژني چې د حمل د پاي ته رسولو وروسته د کوم مشکل لپاره روغتیایي مرکز ته تللي وي؟ تا سو د هغه په اړه څه فکر کوي؟

ولې به هغه ښځي کوم چې د حمل د پاي ته رسولو وروسته د کوم مشکل سره مخ کیږی روغتیایي مرکز ته نه ځي؟ ښځي د نورو روغتیایی ستونزو لپاره روغتیایي مرکزو ته ځي، نو ولې د حمل د لاسه ورکولو ورروسته نه ځي؟ دا ستونزه ولي د نورو روغتیایي ستونزو سره توپیر لري؟

ایا تاسو دا سپارښتنه کوي چي یوه ښځه دي و روغتیایي مرکز ته لاړه شي که چیرې هغه د جنین د سقط څخه وروسته ستونزه ولري؟ ولې؟ ولې نه؟

لنډیز

ایا داسي کوم بل څه شته چې تاسو غواړي اضافه یي کړي کوم چې تاسو فکر کوي چې د دې مسلې سره تړاو لري؟

ایا تاسو کومه پوښتنه یا اندیښنه لرئ؟

ستاسو څخه مننه چي موږ ته مو خپل وخت راکړي.

کله چې اړتیا وي نور تحقیق وکړي

- کولاي شي مثال راکړي؟
- کولاي شي خپل نظرنور هم تشریح کړي؟
- کولاي سي نوريي هم تشریح کړي؟
- کوم څه چې تاسو وویل پری پوه نشوم ، کولاي شي روښانه یي کړي؟
- ایا کوم نور څه هم سته چې تاسو یي ددي موضوع په اړه شریکول غواړي؟
- ایا تا سو او نور ددي سره همږغي یاستی ؟ تاسو او نور په دي اړه څه فکر کوي؟
- داسی معلومیږی چه ځینی کسان تاسو سره همغږی نه دی او یا هم شک لری په دی موضوع کی . آیا تاسو کولی شي موضوغ ښه روښانه کړي؟
